# In between: *Gypsy* in *Drosophila melanogaster* Reveals New Insights into Endogenous Retrovirus Evolution

**DOI:** 10.3390/v6124914

**Published:** 2014-12-09

**Authors:** Franck Touret, François Guiguen, Timothy Greenland, Christophe Terzian

**Affiliations:** 1 Rétrovirus et Pathologie Comparée, Unité Mixte de Recherche 754, Institut National de la Recherche Agronomique, Université Claude Bernard Lyon1, Université de Lyon, Unité Mixte de Service 3444, 69367 Lyon Cedex 7, France; E-Mails: franck.touret@univ-lyon1.fr (F.T.); francois.guiguen@univ-lyon1.fr (F.G.); 2 Ecole Pratique des Hautes Etudes, 75014 Paris, France; 3 39 rue Saint Jean, 69005 Lyon, France; E-Mail: timothy.greenland@gmail.com (T.G.)

**Keywords:** *gypsy*, endogenization, piRNA silencing, Drosophila, *Wolbachia*

## Abstract

Retroviruses are RNA viruses that are able to synthesize a DNA copy of their genome and insert it into a chromosome of the host cell. Sequencing of different eukaryote genomes has revealed the presence of many such endogenous retroviral sequences. The mechanisms by which these retroviral sequences have colonized the genome are still unknown, and the endogenous retrovirus *gypsy* of *Drosophila melanogaster* is a powerful experimental model for deciphering this process *in vivo*. *Gypsy* is expressed in a layer of somatic cells, and then transferred into the oocyte by an unknown mechanism. This critical step is the start of the endogenization process. Moreover *gypsy* has been shown to have infectious properties, probably due to its envelope gene acquired from a baculovirus. Recently we have also shown that *gypsy* maternal transmission is reduced in the presence of the endosymbiotic bacterium *Wolbachia*. These studies demonstrate that *gypsy* is a unique and powerful model for understanding the endogenization of retroviruses.

## 1. Introduction

Retroviruses are enveloped RNA viruses that copy their genome into DNA, then insert it into their host cell’s chromosomes as an essential part of their replication cycle. Classical retroviruses, like HIV or HTLV, propagate through extracellular particles that infect fresh cells in the host and ensure transmission to other individuals of the host species. When this cycle involves somatic cells, the replication cycle involves no alteration of the genetic structure of the host species, but if germline cells are infected but remain competent, the viral genetic information can be passed to successive generations and may eventually become a feature of all members of the host species. Such genetic sequences are called endogenous retroviruses and the dynamic process of their acquisition is called viral endogenization (see Box 1). *In silico* analysis of eukaryote genomes has revealed that such endogenous retroviruses are widespread [1].

Box 1 **Endogenization: However could a viral sequence get into my genes?**

For a viral sequence to become an endogenous element of the host’s genome, the germinal cells themselves must suffer viral infection. The pathway from fusion of germinal cells to the birth of viable and reproductively competent offspring is exceedingly complex and finely balanced. Major disruption of function is very likely to result in fatal errors in embryogenesis and the chromosome bearing a newly inserted viral sequence will then not be transmitted to subsequent generations. This may go a long way to explain why the insertion sites of endogenous retroviral elements do not show a totally random pattern. Even if insertion of a retroviral provirus occurs randomly, those sites that cause too much disruption will be eliminated before they can be seen. Population genetic models could then explain diffusion and fixation of an endogenous provirus within a host population and then within a species. The viral genes themselves may be expressed and the resultant peptides can change the dynamic economy of the host cell. This does not preclude all modification of germ cell function, however; some discrete modifications of viral genes may be tolerated or even beneficial. Indeed several examples of “domesticated” retroviral genetic elements now contribute to genome expression in many species—including humans.

Endogenous retroviruses vary in abundance in eukaryote genomes, and pose many interesting questions. What mutations in their genomes permit their co-existence with their hosts? What impact do these sequences have upon the integrity and function of the host genome? How do the host genomes regulate endogenous viral sequences that have conserved their replicative potential?

Important results have recently been obtained from the analysis of human endogenous retroviruses permitting, among other things, the dating of the incursion of human endogenous retroviral families [2], the “revival” of the postulated infectious retroviral ancestor of the most prolific family of human endogenous retroviruses by *in vitro* directed mutagenesis [3] and a demonstration of the retroviral origin of the *syncytin* gene, which plays an essential part in placental morphogenesis [4]. Nevertheless, studies on the crucial first step of “colonization” of gametes by retroviral sequences leading to endogenization are still sparse. One interesting case is the present active invasion of the koala genome by an exogenous virus closely related to the gibbon ape leukemia virus (GALV) [5], which is present in the genome of some, but not all, animals and appears to be actively infecting their germ-line cells. This animal model is obviously not accessible to an experimental approach. In this review we propose that the *gypsy* retroelement is an exceptional model for understanding the mechanisms of retroviral endogenization.

## 2. *Gypsy:* An Errantivirus of *Drosophila melanogaster*

**Figure 1:**
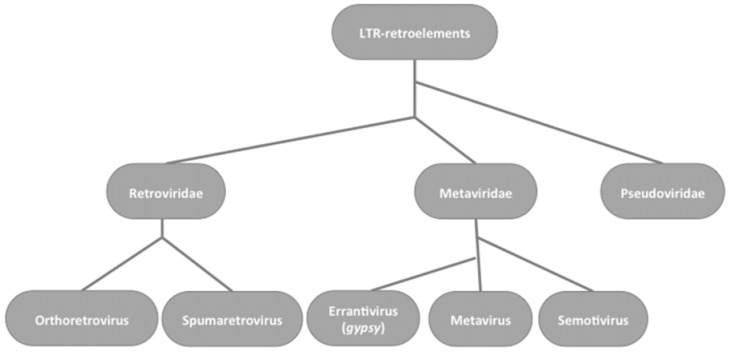
Phylogenetic relationships between LTR-retroelements based on Reverse Transcriptase domains

A helpful model for endogenization and for the balancing regulation needed for stability is provided by the *gypsy* retroelement in *Drosophila melanogaster*. *Gypsy* is classified as an errantivirus, a division of *Metaviridae*, which are a sister group to the *Retroviridae* in the LTR retroelements (Figure 1).

**Figure 2:**
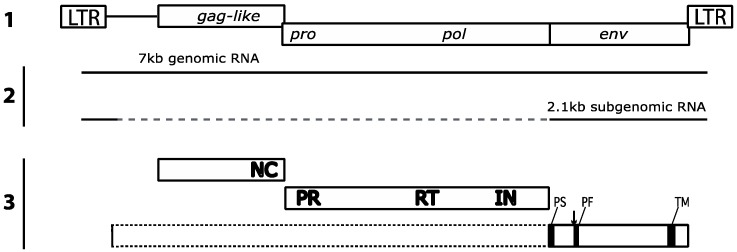
Structure of the *gypsy* proviral genome, mRNAs and proteins. 1- Proviral genetic map: LTR, Long Terminal Repeat. 2- Genomic RNA and spliced env subgenomic RNA. 3- Functional domains of Gag-like, Pro, Pol and Env polyproteins: NC, nucleocapsid; PR, protease; RT, Reverse Transcriptase; IN, Integrase; PS, peptide signal; PF, peptide fusion; TM, transmembrane; arrow indicates the furin cleavage site.

The genome organization of *gypsy* is very similar to that of the classical retroviruses with typical LTRs flanking three open reading frames (ORFs). The first ORF corresponds to a *gag-like* gene with a recognizable NC domain, although the MA and CA domains are unrecognizable, as are those of the HSV spumavirus [6]. Again, like the *Spumaviridae* and some other retroviruses like ASLV or EIAV [7], the *gag* gene lacks a myristilation sequence suggesting a different mechanism for assembly of viral particles at the plasma membrane. The *gag* gene is followed by a recognizable protease (*pro*) and polymerase (*pol*) reading frame, read by a frameshift from the *gag* sequence and by an envelope gene (*env*) expressed from a spliced mRNA lacking the *gag* and *pro-pol* sequences (Figure 2).

Interestingly, it should be noted that *gag*- and *env*-related sequences were identified in Drosophila genomes [8,9] mirroring viral gene domestication events described in vertebrates. However, the cellular roles of these Drosophila putative proteins remain to be demonstrated.

Several gypsy *cis*-regulatory sequences are known. Gypsy contains two internal ribosome entry sites (IRES) [10] and two distinct primer binding sites at the 5’ and 3’ ends of its genome [6]. An unusual characteristic of *gypsy* is that it contains 12 repeats of a sequence in its 5’UTR region which binds the chromatin insulator protein Suppressor of Hairy-Wing [11]. This insulator has the property of completely or partially blocking the activity of enhancers when it occurs between the enhancer and the promoter of a gene. This indicates the effect of *gypsy* proviruses in gene regulatory pathways. Nevertheless, other crucial *cis*-regulatory sequences remain unknown: for example, the nucleo-cytoplasmic export signal for the full-length unspliced gypsy genomic RNA, and the psi packaging RNA element necessary for its encapsidation.

### 2.1. Gypsy Regulation by Flamenco

**Figure 3:**
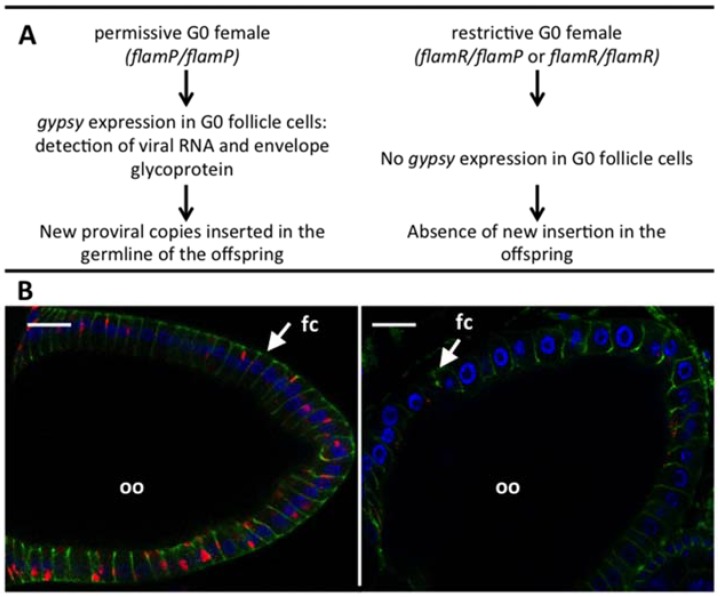
(**A**) Maternal control of gypsy replication by flamenco; (**B**) Immunostaining against gypsy envelope in permissive (left) and restrictive (right) egg chambers (scale bar 20 µm). Blue: Dapi; red: gypsy Env; green: Dlg, a cellular protein present at the junctions between follicle cells. fc: follicle cell; oo: oocyte.

All individuals of *Drosophila melanogaster* carry defective *gypsy* proviruses in the centromeric regions of their X chromosome [12], and certain individuals from wild populations also carry euchromatic integrated *gypsy* proviruses [13]. The number of these novel insertions varies from one to five, and their integration sites vary, suggesting that they represent new germline integrations of an active circulating virus. In addition some laboratory strains of *D. melanogaster* carry a large number (around twenty) of *gypsy* proviruses, and show a high rate of mutation due to the disruption of cellular genes by these mobile proviruses [14]. Genetic analysis of these flies identified a locus named *flamenco* in the heterochromatic region of the X chromosome, which provided a maternal regulation of *gypsy* expression [15]. In permissive flies (*flamP*/*flamP* homozygotes) *gypsy* elements multiply unrestricted, but they are controlled in the presence of the *flamR* dominant restrictive allele. The restriction occurs in a special tissue of the gonads: the follicular cells which are of somatic origin and which surround the oocyte in Drosophila ovary (Figure 3). 

Immuno-labeling shows the polarized expression of *gypsy* Env antigens on the surface of the follicular cells in proximity to the oocyte in permissive samples and its absence in those from flies of the restricted phenotype, correlating with the acquisition of new *gypsy* proviruses in the permissive flies. It should be noted that other Drosophila errantiviruses show the same tissue expression and are similarly controlled from the same locus (Table 1).

**Table 1:** Names and Flybase IDs of errantiviruses regulated by *flamenco* in *Drosophila melanogaster* follicle cells.

Errantivirus	FlyBase ID
*17.6*	FBte0000109
*297*	FBte0000675
*gtwin*	FBte0001062
*gypsy*	FBte0000021
*gypsy4*	FBte0000688
*gypsy5*	FBte0000308
*gypsy6*	FBte0001175
*Idefix*	FBte0000104
*Quasimodo*	FBte0000640
*rover*	FBte0000692
*springer*	FBte0000333
*ZAM*	FBte0000217

The mechanism of transfer does not, however, involve Env directly, because a *gypsy* provirus lacking Env is transferred to oocytes in permissive flies and integrates copies to progeny at a rate similar to that of the intact provirus [16]. The authors have verified absence of *gypsy* Env products in this strain, but cannot rule out presence of a heterologous envelope protein able to pseudotype the *env* defective *gypsy*. Another Drosophila retroelement called ZAM has a similar replicative cycle to *gypsy* [17] and “hijacks” the vitellogenin pathways to enter oocytes [18].

### 2.2. The Genetic “Music” of flamenco

**Figure 4:**
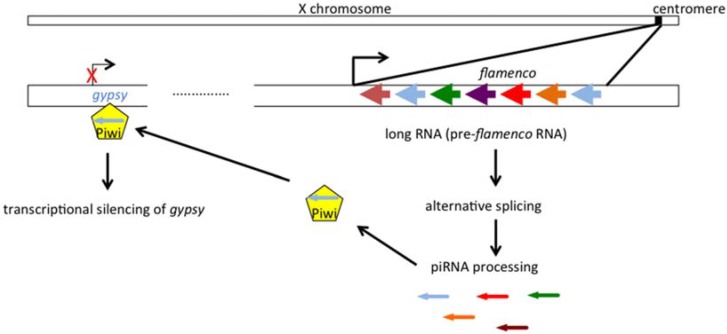
Transcriptional silencing of *gypsy* by *flamenco*. The antisense piRNAs are processed from the pre-flamenco RNA (arrows, colors identify truncated errantivirus, blue=*gypsy*), then loaded onto Piwi. This piRNA-inducing silencing complex establish a repressive chromatin state on *gypsy*, inducing transcriptional silencing.

The *flamenco* locus is located at the heterochromatic 20A locus (X chromosome) and contains numerous co-orientated defective sequences of *gypsy* and other transposable elements. It is not a classical gene that directs the production of a conventional mRNA, but can generate a long non-coding RNA containing many transposable element truncated sequences in an anti-sense direction [19,20]. The transcription is implemented by RNA pol II, is regulated by the transcription factor *cubitus interruptus* [21] and generates a number of different RNA precursors by differential splicing. The precursors are exported to a perinuclear region in follicular cells near to the yb bodies and called flam bodies [22], where they are processed into 25–27 nt fragments which are loaded onto the Piwi protein under control by the co-chaperone Shutdown protein [23], forming piRNA-inducing silencing complex (piRISC). It was first proposed that piRISC was able to target and to cleave sense transcripts from active errantiviruses like *gypsy*, inducing gene silencing at the post-transcriptional level [24]. However several recent results strongly suggest that this complex is imported to the nucleus and silences transposon transcription by establishing H3K9me3 heterochromatic marks [25,26,27,28] (Figure 4).

This mechanism of errantivirus regulation by *flamenco* is called primary piRNA-mediated transcriptional gene silencing (TGS) and operates only in somatic follicular cells; another type of piRNA pathway involving amplification by the “ping pong loop” is active in the germline [29] but does not concern *gypsy* and is not further discussed here. Genesis of *flamenco*-like clusters is a fascinating question, and recent results concerning the dynamic of *flamenco* alleles in Drosophila reveal recurrent insertions and deletions of transposon sequences at the *flam* locus [30].

## 3. *Gypsy:* An Endogenous Retrovirus with Infectious Properties

The *gypsy* element in Drosophila provides a good experimental system for the various steps in endogenization. In order to invade the genome of a new species, the potential endogenous element must first establish itself in at least one individual of that species. *Gypsy* is an errantivirus, a subtype of the *Metaviridae* division of LTR transposons, which possess a third ORF coding for an *env-like* gene. This might permit classical retrovirus-like infectivity.

To test this, Kim *et al.* developed an experimental system where culture media including homogenized *gypsy*-expressing Drosophila were fed to permissive larvae lacking active *gypsy* proviral sequences. A highly-selective genetic test allowed an estimation of the frequency of transfer of new proviral copies into the germline of progeny by the observations of mutations caused by insertion of a *gypsy* provirus into the *ovo* locus [31]. Similar results were obtained by Song *et al.* using purified *gypsy* particles from permissive adult females; they also showed that infection was abrogated by pre-treatment of the particles by an anti-Env antibody, suggesting an active role for *gypsy* Env in the infection process [32].

**Figure 5:**
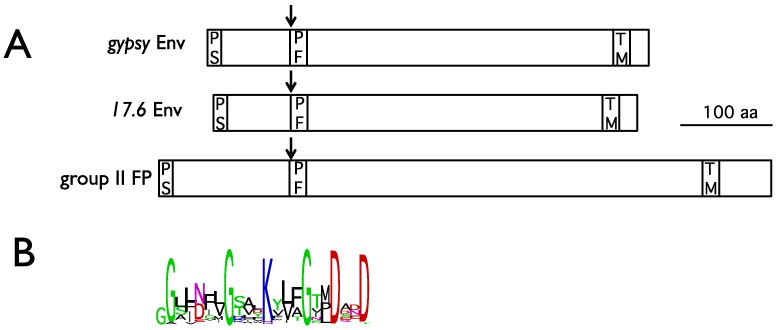
(**A**) comparison of *gypsy* Env , 17.6 Env and baculovirus group II Fusion Protein (FP) structural properties. Arrows indicate the furin cleavage site. PS, peptide signal; PF, peptide fusion; TM, transmembrane. (**B**) Logo representation (weblogo.berkeley.edu/logo.cgi) of the peptide fusion consensus sequence between 23 errantiviruses Env and 9 group II FP amino acid sequences.

A defining characteristic of the errantiviruses, as compared to the metaviruses and semotiviruses, is that they possess a third open reading frame coding for an envelope glycoprotein and expressed from a sub-genomic spliced mRNA similar to that of vertebrate retroviruses [33]. The *gypsy* Env is atypical for retroviruses, but shows significant homology to the baculovirus fusion protein FP [34] and was probably “captured” by insertion of a LTR retrotransposon, which lacks the *env* gene, into the dsDNA genome of a baculovirus infecting the host cell. Baculoviruses have a replication strategy and a cellular tropism quite different to those of the errantiviruses. Baculovirus particles exist in two different forms: the occlusion derived viruses (ODV) and the budded viruses (BV). ODV are released from occlusion bodies, and initiate intestinal infection after ingestion by the insect host, whereas BV buds out of infected cells and mediate cell-to-cell spread throughout the insect. BV and ODV differ mainly in the origin of their envelopes. The errantiviral envelope shows similarities with the FP envelope protein of the BV particles present in the group II nucleopolyhedroviruses, which allows cell penetration by a pH-dependent fusion. This similarity is highly significant for the peptide fusion domain, present in all viral fusion proteins of class I (Figure 5) [35] and which allows fusion between the virus and the target cell membrane [36]. *In silico* analysis predicts, however, only a weak fusion potential for these peptides, in comparison to that of the HRSV paramyxovirus [37].

## 4. *Wolbachia* Influences *Gypsy* Maternal Transmission

We have been considering the overall host and parasite context of the interactions between *gypsy* and Drosophila and have recently concentrated upon another maternally-transmitted agent in Drosophila: *Wolbachia*, which passes from mother to offspring in the oocyte cytoplasm [38]. Interestingly, *Wolbachia* reduces the replication of several exogenous RNA viruses [39,40] and we have shown that, in the presence of one *Wolbachia* variant (*w*Mel), which is at present becoming a major strain in *Drosophila melanogaster* [41], the maternal transmission of *gypsy,* as measured by its insertion into the *ovo* gene, is substantially reduced [42]. This diminution does not involve *flamenco* restriction, because rates of *gypsy* insertion do not differ between *w*Mel+ and *w*Mel− *flamenco* restrictive flies. We hypothesize that *Wolbachia* competes efficiently with *gypsy* for a posterior position within the oocyte, and thereby impedes *gypsy* maternal transmission into the offspring germline cells. We are considering whether this mechanism operates for other endogenous retroviruses of Drosophila, or indeed for other cases of maternal transmission of exogenous viruses.

## 5. Conclusions

**Figure 6:**
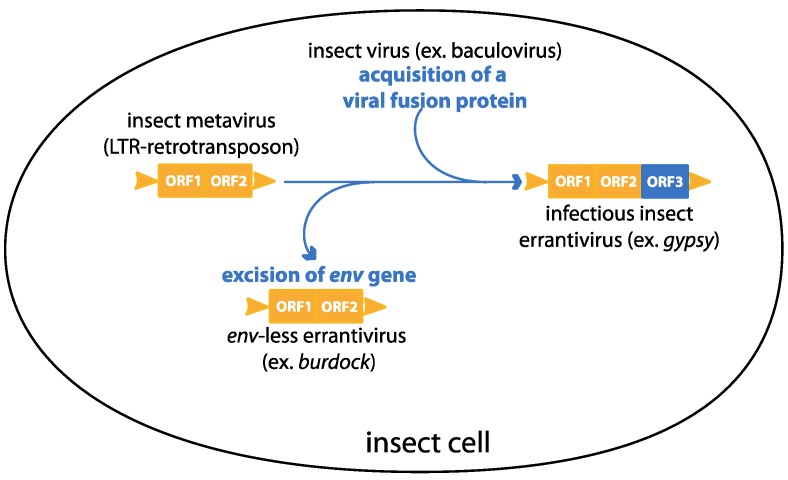
A model describing the dynamic of acquisition and excision of an *env* gene in errantiviruses

*Gypsy* is a relevant *in vivo* model of endogenization because of its natural presence in *Drosophila melanogaster* for which powerful molecular and genetic tools are available. The fact that it is quite easy to induce *gypsy* mobilization and transfer to the germline makes it a unique model that allows the study of endogenous retrovirus genesis but also of a mechanism of envelope gene procurement, making *gypsy* an excellent model of “exogenization” process. The acquisition or loss of an envelope gene can be seen as a dynamic system showing an unstable equilibrium between a LTR retrotransposon lacking the envelope gene and an infectious retrovirus (Figure 6).

Identification of an errantivirus, *burdock*, closely related to *gypsy* but lacking an envelope gene reinforces this hypothesis [43]. However, the precise role of *gypsy* Env remains an open question. We have obtained some preliminary results that suggest that Env is incorporated into *gypsy* particles produced in a Drosophila cell culture line, which makes this *in vitro* tool suitable for Env analysis.

*Gypsy* and the errantiviruses represent a hybrid type of viral element, which resembles a retroelement in its replication enzymatic machinery, but has opportunistically acquired a viral gene coding for a protein with fusogenic properties. The finding that *gypsy* also interacts with *Wolbachia* provides the first evidence that a novel factor like an endosymbiotic bacterium can influence colonization of the genome by retroelements.

## References

[1] Katzourakis A., Gifford R.J. (2010). Endogenous viral elements in animal genomes. PLOS Genet.

[2] Blikstad V., Benachenhou F., Sperber G.O., Blomberg J. (2008). Evolution of human endogenous retroviral sequences: A conceptual account. Cell. Mol. Life Sci..

[3] Dewannieux M., Harper F., Richaud A., Letzelter C., Ribet D., Pierron G., Heidmann T. (2006). Identification of an infectious progenitor for the multiple-copy HERV-K human endogenous retroelements. Genome Res..

[4] Mallet F., Bouton O., Prudhomme S., Cheynet V., Oriol G., Bonnaud B., Lucotte G., Duret L., Mandrand B. (2004). The endogenous retroviral locus ERVWE1 is a bona fide gene involved in hominoid placental physiology. Proc. Natl. Acad. Sci. USA.

[5] Tarlinton R.E., Meers J., Young P.R. (2006). Retroviral invasion of the koala genome. Nature.

[6] Gabus C., Ivanyi-Nagy R., Depollier J., Bucheton A., Pelisson A., Darlix J.-L. (2006). Characterization of a nucleocapsid-like region and of two distinct primer tRNALys,2 binding sites in the endogenous retrovirus Gypsy. Nucleic Acids Res..

[7] Delelis O., Lehmann-Che J., Saïb A. (2004). Foamy viruses—A world apart. Curr. Opin. Microbiol..

[8] Malik H.S., Henikoff S. (2005). Positive selection of *Iris*, a retroviral *envelope*–derived host gene in *Drosophila melanogaster*. PLOS Genet..

[9] Nefedova L.N., Kuzmin I.V., Makhnovskii P.A., Kim A.I. (2014). Domesticated retroviral GAG gene in Drosophila: New functions for an old gene. Virology.

[10] Ronfort C., de Breyne S., Sandrin V., Darlix J.-L., Ohlmann T. (2004). Characterization of two distinct RNA domains that regulate translation of the Drosophila gypsy retroelement. RNA.

[11] Geyer P.K., Corces V.G. (1992). DNA position-specific repression of transcription by a *Drosophila* zinc finger protein. Genes Dev..

[12] Lambertsson A., Andersson S., Johansson T. (1989). Cloning and characterization of variable-sized gypsy mobile elements in *Drosophila melanogaster*. Plasmid.

[13] Biémont C., Lemeunier F., Garcia Guerreiro M.P., Brookfield J.F., Gautier C., Aulard S., Pasyukova E.G. (1994). Population dynamics of the copia, mdg1, mdg3, gypsy, and P transposable elements in a natural population of *Drosophila melanogaster*. Genet. Res..

[14] Mével-Ninio M., Terracol R., Kafatos F.C. (1991). The ovo gene of Drosophila encodes a zinc finger protein required for female germ line development. EMBO J..

[15] Prud’homme N., Gans M., Masson M., Terzian C., Bucheton A. (1995). Flamenco, a gene controlling the gypsy retrovirus of *Drosophila melanogaster*. Genetics.

[16] Chalvet F., Teysset L., Terzian C., Prud’homme N., Santamaria P., Bucheton A., Pélisson A. (1999). Proviral amplification of the Gypsy endogenous retrovirus of *Drosophila melanogaster* involves env-independent invasion of the female germline. EMBO J..

[17] Leblanc P., Desset S., Giorgi F., Taddei A.R., Fausto A.M., Mazzini M., Dastugue B., Vaury C. (2000). Life cycle of an endogenous retrovirus, ZAM, in *Drosophila melanogaster*. J. Virol..

[18] Brasset E., Taddei A.R., Arnaud F., Faye B., Fausto A.M., Mazzini M., Giorgi F., Vaury C. (2006). Viral particles of the endogenous retrovirus ZAM from *Drosophila melanogaster* use a pre-existing endosome/exosome pathway for transfer to the oocyte. Retrovirology.

[19] Malone C.D., Brennecke J., Dus M., Stark A., McCombie W.R., Sachidanandam R., Hannon G.J. (2009). Specialized piRNA pathways act in germline and somatic tissues of the Drosophila ovary. Cell.

[20] Brennecke J., Aravin A.A., Stark A., Dus M., Kellis M., Sachidanandam R., Hannon G.J. (2007). Discrete small RNA-generating loci as master regulators of transposon activity in Drosophila. Cell.

[21] Goriaux C., Desset S., Renaud Y., Vaury C., Brasset E. (2014). Transcriptional properties and splicing of the flamenco piRNA cluster. EMBO Rep..

[22] Murota Y., Ishizu H., Nakagawa S., Iwasaki Y.W., Shibata S., Kamatani M.K., Saito K., Okano H., Siomi H., Siomi M.C. (2014). Yb integrates piRNA intermediates and processing factors into perinuclear bodies to enhance piRISC assembly. Cell Rep..

[23] Olivieri D., Senti K.-A., Subramanian S., Sachidanandam R., Brennecke J. (2012). The cochaperone shutdown defines a group of biogenesis factors essential for all piRNA populations in Drosophila. Mol. Cell.

[24] Bourc’his D., Voinnet O. (2010). A small-RNA perspective on gametogenesis, fertilization, and early zygotic development. Science.

[25] Sienski G., Dönertas D., Brennecke J. (2012). Transcriptional silencing of transposons by Piwi and maelstrom and its impact on chromatin state and gene expression. Cell.

[26] Wang S.H., Elgin S.C.R. (2011). Drosophila Piwi functions downstream of piRNA production mediating a chromatin-based transposon silencing mechanism in female germ line. Proc. Natl. Acad. Sci. USA.

[27] Le Thomas A., Rogers A.K., Webster A., Marinov G.K., Liao S.E., Perkins E.M., Hur J.K., Aravin A.A., Tóth K.F. (2013). Piwi induces piRNA-guided transcriptional silencing and establishment of a repressive chromatin state. Genes Dev..

[28] Rozhkov N.V., Hammell M., Hannon G.J. (2013). Multiple roles for Piwi in silencing Drosophila transposons. Genes Dev..

[29] Han B.W., Zamore P.D. (2014). piRNAs. Curr. Biol..

[30] Zanni V., Eymery A., Coiffet M., Zytnicki M., Luyten I., Quesneville H., Vaury C., Jensen S. (2013). Distribution, evolution, and diversity of retrotransposons at the flamenco locus reflect the regulatory properties of piRNA clusters. Proc. Natl. Acad. Sci. USA.

[31] Kim A., Terzian C., Santamaria P., Pelisson A., Purd’homme N., Bucheton A. (1994). Retroviruses in invertebrates: The gypsy retrotransposon is apparently an infectious retrovirus of Drosophila melanogaster. Proc. Natl. Acad. Sci. USA.

[32] Song S.U., Gerasimova T., Kurkulos M., Boeke J.D., Corces V.G. (1994). An env-like protein encoded by a Drosophila retroelement: Evidence that gypsy is an infectious retrovirus. Genes Dev..

[33] Pelisson A., Song S.U., Prud’homme N., Smith P.A., Bucheton A., Corces V.G. (1994). Gypsy transposition correlates with the production of a retroviral envelope-like protein under the tissue-specific control of the Drosophila flamenco gene. EMBO J..

[34] Rohrmann G.F., Karplus P.A. (2001). Relatedness of baculovirus and gypsy retrotransposon envelope proteins. BMC Evol. Biol..

[35] Garry C.E., Garry R.F. (2008). Proteomics computational analyses suggest that baculovirus GP64 superfamily proteins are class III penetrenes. Virol. J..

[36] Gallaher W.R., DiSimone C., Buchmeier M.J. (2001). The viral transmembrane superfamily: Possible divergence of Arenavirus and Filovirus glycoproteins from a common RNA virus ancestor. BMC Microbiol..

[37] Misseri Y., Labesse G., Bucheton A., Terzian C. (2003). Comparative sequence analysis and predictions for the envelope glycoproteins of insect endogenous retroviruses. Trends Microbiol..

[38] Ferree P.M., Frydman H.M., Li J.M., Cao J., Wieschaus E., Sullivan W. (2005). Wolbachia utilizes host microtubules and dynein for anterior localization in the Drosophila oocyte. PLOS Pathog..

[39] Hedges L.M., Brownlie J.C., O’Neill S.L., Johnson K.N. (2008). Wolbachia and virus protection in insects. Science.

[40] Teixeira L., Ferreira Á., Ashburner M. (2008). The bacterial symbiont Wolbachia induces resistance to RNA viral infections in *Drosophila melanogaster*. PLoS Biol..

[41] Riegler M., Sidhu M., Miller W.J., O’Neill S.L. (2005). Evidence for a global Wolbachia replacement in *Drosophila melanogaster*. Curr. Biol..

[42] Touret F., Guiguen F., Terzian C. (2014). Wolbachia influences the maternal transmission of the gypsy endogenous retrovirus in *Drosophila melanogaster*. MBio.

[43] Terzian C., Pélisson A., Bucheton A. (2001). Evolution and phylogeny of insect endogenous retroviruses. BMC Evol. Biol..

